# Rotational Thromboelastometry as a Diagnostic Tool for Persistent Infection in Two-Stage Exchange Arthroplasty

**DOI:** 10.3390/jcm13071942

**Published:** 2024-03-27

**Authors:** Andreas G. Tsantes, Dimitrios V. Papadopoulos, Stavros Goumenos, Ioannis G. Trikoupis, Konstantina A. Tsante, Christos Koutserimpas, Panagiotis Koulouvaris, Vasileios Petrakis, Aristeidis G. Vaiopoulos, Daniele Piovani, Georgios K. Nikolopoulos, Andreas F. Mavrogenis, Panayiotis J. Papagelopoulos, Stefanos Bonovas, Argirios E. Tsantes

**Affiliations:** 1 Laboratory of Haematology and Blood Bank Unit, “Attiko” Hospital, School of Medicine, National and Kapodistrian University of Athens, 12462 Athens, Greece; ktsante@yahoo.com (K.A.T.); avaiopoulos@gmail.com (A.G.V.); atsantes@yahoo.com (A.E.T.); 2 Microbiology Department, “Saint Savvas” Oncology Hospital, 11522 Athens, Greece; 3 Second Department of Orthopaedics, School of Medicine, National and Kapodistrian University of Athens, 12462 Athens, Greece; di_papadopoulos@yahoo.gr; 4 First Department of Orthopaedics, School of Medicine, National and Kapodistrian University of Athens, 12462 Athens, Greece; stgoumenos@gmail.com (S.G.); gtrikoupis@hotmail.com (I.G.T.); info@drkoulouvaris.gr (P.K.); afm@otenet.gr (A.F.M.); pjporthopedic@gmail.com (P.J.P.); 5 Orthopaedic Surgery and Sports Medicine Department, Croix-Rousse Hospital, 69004 Lyon, France; chrisku91@hotmail.com; 6 Department of Anatomy, School of Medicine, National and Kapodistrian University of Athens, 11527 Athens, Greece; 7 2nd University Department of Internal Medicine, University General Hospital of Alexandroupolis, Democritus University of Thrace, 68132 Alexandroupolis, Greece; vasilispetrakis1994@gmail.com; 8 HIV Unit, Department of Infectious Diseases, University General Hospital of Alexandroupolis, Democritus University of Thrace, 68132 Alexandroupolis, Greece; 9 Department of Biomedical Sciences, Humanitas University, 20072 Milan, Italy; dpiovani@hotmail.com (D.P.); sbonovas@gmail.com (S.B.); 10 IRCCS Humanitas Research Hospital, 20089 Milan, Italy; 11 Laboratory of Medical Statistics, Epidemiology and Public Health, Medical School, University of Cyprus, Nicosia 1678, Cyprus

**Keywords:** periprosthetic joint infections, persistent infection, two-stage exchange arthroplasty, diagnosis, rotational thromboelastometry

## Abstract

**Background/Objectives:** There is a lack of reliable biomarkers for diagnosis of infection eradication prior to second-stage reimplantation in two-stage exchange arthroplasty for periprosthetic joint infections (PJIs). The aim of this study was to assess the diagnostic accuracy of rotational thromboelastometry (ROTEM) for persistent infection in two-stage exchange arthroplasties. **Methods:** A pilot, retrospective analysis was performed including 70 patients who underwent a two-stage exchange arthroplasty for PJI. They were categorized as patients without (*n* = 64) or patients with persistent infection (*n* = 6) prior to reimplantation. Definition of persistent infection prior to reimplantation was based on the 2018 ICM criteria. Conventional coagulation biomarkers and ROTEM parameters were compared between groups. **Results:** Higher FIBTEM MCF values were associated with persistent infection (odds ratio [OR], 1.30, 95% confidence interval [CI], 1.04–1.63; *p* = 0.020), and FIBTEM MCF had the highest diagnostic accuracy for persistent infection prior to second-stage reimplantation (AUC, 0.907; 95% CI, 0.812–1.000). A cut-off value ≥ 18 mm for FIBTEM MCF was found to have 100.0% sensitivity and 73.4% specificity for diagnosing persistent infection prior to second-stage reimplantation. Moreover, the diagnostic accuracy of FIBTEM MCF was higher than that of fibrinogen levels (*p* = 0.036) and D-dimer (*p* = 0.006). **Conclusions:** Our findings indicate that ROTEM parameters have the potential to identify persistent infections before reimplantation in two-stage exchange arthroplasties for PJI. Such coagulation biomarkers could provide guidance regarding the optimal timing for reimplantation. Further studies in larger populations are warranted to validate the diagnostic accuracy of ROTEM parameters for persistent PJI.

## 1. Introduction

The number of periprosthetic joint infections (PJIs) has been rising over the past decades, with a reported rate of 1–4% following total knee and hip arthroplasties [1,2]. The preferred strategy for the treatment of these infections involves a two-stage exchange arthroplasty; the infected implants are removed and replaced by antibiotic spacers during the first stage, followed by a period of systemic antibiotics, while reimplantation of the final implants is performed as a second stage, usually 2–3 months later. The rationale for this approach is that complete eradication of the infection should be achieved prior to reimplantation through a thorough irrigation and debridement during the first stage, followed by a subsequent course of antibiotics. However, despite the advances in the treatment strategy of these infections, the reported rate of reinfections is still high, estimated to be up to 30% [3,4].

Blood biomarkers with high sensitivity and specificity for diagnosing persistent infections prior to second-stage reimplantation would be of great value as this would guide surgeons regarding the optimal time for proceeding to the second stage. However, diagnosis of persistent infection is challenging since the accuracy of the available set of biomarkers is low [5,6,7,8]. Although joint aspiration and synovial fluid evaluation is considered the most reliable method for diagnosis of PJI, there are certain drawbacks to this approach; in many cases there are false negative results, especially in low-grade infections, while there are often dry taps without any synovial fluid for evaluation. Moreover, serum inflammatory markers such as erythrocyte sedimentation rate (ESR) and C-reactive protein (CRP) can remain elevated, even in proven cases of infection eradication; thus, their diagnostic accuracy is limited [9].

Although there is a substantial body of literature linking infection and its associated inflammatory process with a hypercoagulable state, studies regarding the relationship between hemostatic derangements and PJI have been sparse until now [10,11,12]. Rotational thromboelastometry (ROTEM) is a laboratory approach that evaluates the coagulation mechanism through a thorough analysis of the viscoelastic properties of whole blood specimens. Compared to conventional coagulation studies, ROTEM analysis has the advantage of evaluating all phases of hemostasis from clot formation to clot breakdown, while it has been shown to have a high diagnostic accuracy for detecting coagulation derangements that are associated with various pathologies such as thromboembolic complications and excessive bleeding [13].

The aim of this study was to examine whether ROTEM parameters are associated with persistent joint infections prior to second-stage reimplantation, and also to investigate the diagnostic accuracy of these parameters to detect persistent PJI.

## 2. Methods

A pilot, retrospective study was performed including patients who were diagnosed with PJI following total knee or hip arthroplasty and underwent a two-stage exchange arthroplasty between May 2019 and July 2022. Patients were evaluated following the index arthroplasty due to pain, local signs of inflammation such as erythema, or wound discharge and sinus tract development. Evaluation of these patients included imaging studies (X-rays), laboratory assessment (complete blood count and inflammatory markers), and joint aspiration for evaluation of the synovial fluid. The diagnosis of PJI was based on the criteria of the International Consensus Meeting (ICM) for PJI in 2018 [14]. Patients with coagulopathies or infections in other organs were excluded from the study. Based on our treatment protocol for PJI, implants were removed, a thorough irrigation and debridement was performed, and spacers were placed during the first stage, while all patients received prophylactic anticoagulation for 4 weeks and antibiotics for 6–8 weeks, postoperatively. A two-week antibiotic holiday period was maintained before second-stage reimplantation in all patients. Patients received antibiotics based on the pathogen that was isolated during the first stage. The most commonly isolated pathogen was *S. aureus*, and the most common antibiotics included vancomycin and clindamycin. The time of second-stage reimplantation was decided based on the following criteria: a completely healed wound, no pain or any clinical sign of infection, and a downward trend in serologic biomarkers (ESR and CRP) after the first stage. These markers had to be within the normal range or close to it. Moreover, 5 synovial tissue samples for histological analysis and culture were collected during the second-stage reimplantation in all patients. The culture mediums for the synovial tissue samples included blood agar and MacConkey agar, while cultures were kept for 14 days before being declared negative.

The study population was categorized into two groups: patients with persistent infection during the second-stage reimplantation (Group A) and patients without persistent infection (Group B). Persistent infection was defined as an infection diagnosed intraoperatively during the second-stage reimplantation based on the ICM 2018 intraoperative criteria for definite infection (positive histology and positive purulence with or without positive culture) or inconclusive infection (at least a single positive culture and positive histology and/or positive purulence), or as a new PJI based on the ICM 2018 that occurred during the first postoperative year. Demographics, laboratory findings, and clinical characteristics were recorded for each patient. The study received approval by the hospital’s Institutional Review Board (Ref number: 199/23-03-2023), while all patients provided informed consent.

Laboratory assessment of the study population was performed prior to reimplantation and included conventional coagulation studies (prothrombin time [PT], activated partial thromboplastin time [aPTT], fibrinogen, D-dimer) and ROTEM analysis (Supplemental Methods). For the ROTEM analysis, whole blood samples were analyzed in a ROTEM analyzer (delta ROTEM, Tem Innovation GmbH, Munich, Germany). All samples were analyzed within 90 min from the blood draw. ROTEM analysis included EXTEM, INTEM, and FIBTEM assays for evaluation of the extrinsic coagulation pathway, the intrinsic coagulation pathway, and the contribution of fibrinogen in coagulation, respectively. The ROTEM parameters that were evaluated included the coagulation time (CT, s) indicating the time from the beginning of the analysis to the formation of a clot of 2 mm in amplitude; the clot formation time (CFT, s) which is the time from CT to formation of a clot of 20 mm in amplitude; the clot amplitude at 10 min (A10, mm); the alpha angle (a°), which is the angle between the horizontal line (x-axis) and the tangent to the ROTEM trace at 2 mm clot amplitude; the maximum clot firmness (MCF, mm), which reflects the maximum clot amplitude; and the lysis index at 60 min (LI60, %), which is the percentage of the residual clot firmness at 60 min compared to MCF, indicating the fibrinolysis speed.

### Statistical Analysis

Study data are presented as mean ± standard deviation (SD), median and interquartile range (IQR), or percentage when appropriate. The non-parametric Wilcoxon rank-sum test and the chi-squared test were used to compare parameters between groups. Moreover, in order to evaluate the independent association between the coagulation state, as reflected by ROTEM parameters, and development of persistent infections, a multivariable logistic regression analysis was performed with persistent infection as the dependent variable, and the Body Mass Index (BMI), gender, age, smoking status, Charlson comorbidity index, chronic anticoagulants, and ROTEM results as independent variables. Moreover, the diagnostic accuracy of ROTEM parameters to detect persistent infections was assessed using receiver operating characteristic (ROC) curves and their respective areas under these ROC curves (AUC). To identify the optimal cut-off value for each ROTEM parameter, the Youden Index was used. Finally, the Hanley and McNeil method was used to compare the diagnostic accuracies of ROTEM parameters and conventional coagulation biomarkers [15]. Two-tailed *p* < 0.05 was considered to indicate statistical significance. Stata statistical software, version 15, was used for analysis.

## 3. Results

**Figure 1:**
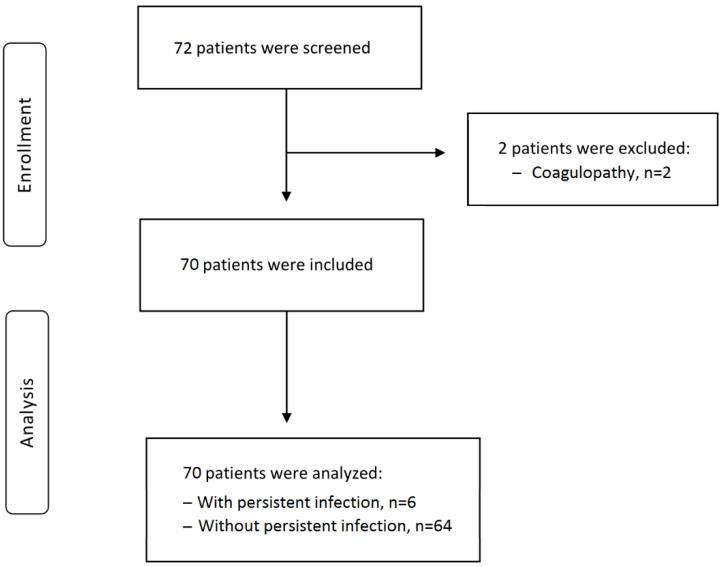
Flowchart of the study population.

Seventy-two patients were considered eligible for the study. Two were excluded because they had congenital coagulopathy (Factor V Leiden), and 70 patients were finally included in the study (Figure 1).

There were 6 patients with persistent infection (Group A) and 64 without (Group B). Among the six patients with persistent infection, for two the diagnosis was made intraoperatively during the second stage (positive intraoperative cultures and positive histology), while the remaining four developed a new PJI during the postoperative period. The isolated pathogens in these six patients with persistent infection included methicillin resistant *Staphylococcus aureus* (*n* = 2), Coagulase-negative Staphylococcus species (*S. epidermidis n* = 2 and *S. lugdunensis n* = 1), and Enterococcus species (*n* = 1). Patients with or without persistent infections did not differ in terms of age (median: 64.5 vs. 70 years, *p* = 0.014), gender (males: 50.0% vs. 42.1%, *p* = 0.51), smoking status (33.0% vs. 6.2% smokers, *p* = 0.07), BMI (22.5 vs. 24.0 kg/m^2^, *p* = 0.28), or use of chronic anticoagulants (33.3% vs. 25.0%, *p* = 0.48; Table 1).

The results of the factors of the conventional coagulation studies, such as platelets, PT, aPTT, and D-dimer, were similar between patients with or without persistent infection (Table 2). However, fibrinogen levels were higher in patients with persistent infection (median: 413.0 vs. 330.0 mg/dL, *p* = 0.0029; Table 2). Regarding the inflammatory markers, patients with and without persistent infection had comparable CRP (medians: 5.4 vs. 3.9 mg/L, *p* = 0.31) and ESR (medians: 15.5 vs. 12.0 mm/h, *p* = 0.38) levels.

### 3.1. ROTEM Parameters and Persistent Infection

The ROTEM parameters A10 and MCF differed between patients with or without persistent infection. Specifically, EXTEM A10, INTEM A10, and FIBTEM A10 were higher in patients with persistent infection (EXTEM A10, median: 61 vs. 52 mm, *p* = 0.030; INTEM A10: 63 vs. 53 mm, *p* = 0.004; and FIBTEM A10: 17.5 vs. 11 mm, *p* = 0.023), while EXTEM MCF, INTEM MCF, and FIBTEM MCF were also higher in patients with persistent infection (EXTEM MCF, median: 73 vs. 62 mm, *p* = 0.010; INTEM MCF: 70.5 vs. 60.5 mm, *p* = 0.006; and FIBTEM MCF: 22.5 vs. 13.5 mm, *p* = 0.001; Table 3). An increased clot strength and thrombin formation in patients with persistent infection was found, as indicated by the higher MCF values.

The association between the higher coagulation potential as reflected by ROTEM findings and persistent infection was also revealed by the findings of the multivariable logistic regression analysis (Table 4). Specifically, persistent infection was associated with higher INTEM and FIBTEM A10 (INTEM A10: OR, 1.22, 95% CI, 1.02–1.46; *p* = 0.030, and FIBTEM A10: OR, 1.29, 95% CI, 1.004–1.66; *p* = 0.044), and higher EXTEM, INTEM, and FIBTEM MCF (EXTEM MCF: OR, 1.21, 95% CI, 1.02–1.43; *p* = 0.026, INTEM MCF: OR, 1.27, 95% CI, 1.02–1.57; *p* = 0.026, and FIBTEM MCF: OR, 1.30, 95% CI, 1.04–1.63; *p* = 0.020).

### 3.2. Diagnostic Accuracy of ROTEM Parameters and Fibrinogen Levels for Persistent Infection

Among ROTEM parameters, FIBTEM MCF had the highest diagnostic accuracy for persistent infection prior to second-stage reimplantation (AUC, 0.907; 95% CI, 0.812–1.000), followed by INTEM A10 (AUC, 0.856; 95% CI, 0.745–0.967) and INTEM MCF (AUC, 0.837; 95% CI, 0.701–0.973; Table 5). The cut-off value ≥ 18 mm for FIBTEM MCF was found to have 100.0% sensitivity and 73.4% specificity for diagnosing persistent infections prior to second-stage reimplantation.

**Figure 2:**
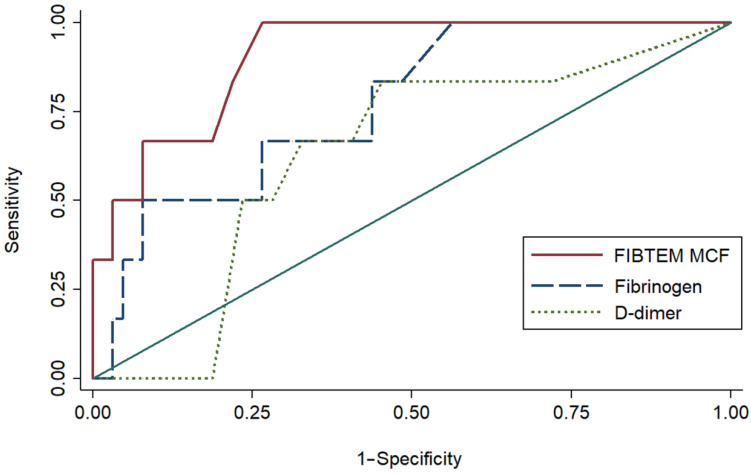
The areas under the receiver operating characteristic curve of FIBTEM MCF, fibrinogen levels, and D-dimer levels for diagnosis of persistent infection prior to reimplantation.

The diagnostic accuracy of FIBTEM MCF was similar to that of INTEM A10 (*p* = 0.48) and INTEM MCF (*p* = 0.35), while it was higher than that of fibrinogen levels (*p* = 0.036), and D-dimer (*p* = 0.006; Figure 2). Finally, the diagnostic accuracy of FIBTEM MCF was found to be higher than that of CRP (*p* < 0.001) and ESR (*p* = 0.005).

## 4. Discussion

Several parameters, such as synovial fluid cultures, synovial white blood cell count, and inflammatory markers, have been evaluated for their diagnostic accuracy in detecting persistent joint infections prior to second-stage reimplantation [6,7,8]. However, due to the low diagnostic accuracy of these biomarkers, the search for the best diagnostic approach is still underway. Based on the established close association between coagulation abnormalities and infection, development of new coagulation-related biomarkers may be promising for the identification of those cases in which infection eradication has not been achieved. In line with this, the findings of this pilot study indicate that ROTEM parameters have the potential to identify those cases of PJI following hip and knee arthroplasties in which the first stage of revision surgery did not achieve eradication of the infection. Specifically, a higher MCF value was associated with a higher risk of persistent joint infection, indicating that persistent infections are related to a hypercoagulable state in which clot strength is increased. Moreover, FIBTEM MCF was found to have the best detection accuracy for persistent infections among all ROTEM parameters, with a FIBTEM value ≥ 18 mm having 100% sensitivity and 73.3% specificity for detecting persistent infections.

The association between the inflammatory process involved in infections and a prothrombotic state been shown in several studies [12,16]. Several pathogenetic mechanisms are involved in this prothrombotic state, including extrinsic pathway activation, cytokine-induced coagulation amplification, and fibrinolysis inhibition. However, as opposed to the prothrombotic state with which less severe infections are associated, severe sepsis is associated with a hypocoagulable state. This was demonstrated in 2011 by Adamzik et al. [17]. In their study, Admazik et al. compared several ROTEM parameters between survivors and non-survivors following severe sepsis, and the authors found significant differences in CFT, MCF, and α angle between survivors and non-survivors, with non-survivors showing prolonged CFT, reduced MCF, and reduced α angle. Moreover, defining thromboelastometry values as normal or pathological based on median cut-offs, the study found a 30-day survival rate of 85.7% when all thromboelastometry variables were normal, dropping to 58.7% with at least one pathological variable. Multivariate analysis showed that the presence of at least one pathological thromboelastometry variable was a better predictor of 30-day survival than the SAPS II and SOFA scores, highlighting the significance of the coagulation system in the prognosis of sepsis.

In orthopedics, Saxena et al. first described the effects of PJI on hemostasis [18]. The authors of this study highlighted that infecting pathogens cause the release of multiple inflammatory mediators resulting in overexpression of factor VIIa, while bacterial toxins lead to direct activation of the coagulation cascade through direct endothelial injury. These findings are also supported by the results of our study, since higher MCF, which reflects enhanced thrombin formation, was strongly associated with development of PJI. Another interesting finding of our study is that FIBTEM parameters that reflect the contribution of fibrinogen to coagulation were found to have the best diagnostic accuracy for persistent infections compared to EXTEM and INTEM parameters. This could be explained by the key role of fibrinogen as an activator and mediator of the inflammation process. Fibrinogen has a strong proinflammatory function, which is mediated through its binding to several receptors, such as the CD11b/CD18 integrin receptor on macrophages and other immune cells. This binding results in the release of various inflammatory cytokines such as tumor necrosis factor (TNF)-α or IL-1β, which in turns leads to activation of the inflammatory cascade [19]. The strong association between altered FIBTEM parameters and infection has been also shown in other studies evaluating patients in various clinical settings, such as in bacterial sepsis or in COVID-19 infection, highlighting the prominent role of fibrinogen in infection-related coagulation derangements [20,21].

There are only a few studies evaluating the role of coagulation-related biomarkers such as fibrinogen and D-dimer in the diagnosis of persistent joint infections following the first stage of revision hip and knee arthroplasties [22,23,24]. Bin et al. assessed the preoperative fibrinogen levels prior to second-stage final reimplantation in patients who underwent a two-stage exchange arthroplasty to investigate whether they differed between those with and without positive cultures at the second stage [23]. The authors found that preoperative fibrinogen levels prior to the second stage were below the cut-off level (360 mg/dL) for PJI in those patients in whom intraoperative cultures were negative during the reimplantation. This is in line with our results, since the median value of fibrinogen in our patients without persistent infection was also below this cut-off value (334 mg/dL). However, Bin et al. did not evaluate the diagnostic accuracy of fibrinogen levels for persistent infections. The diagnostic accuracy of fibrinogen for persistent infection prior to reimplantation was also evaluated in another study by Xu et al. [24]. The authors reviewed 129 hips treated with two-stage exchange arthroplasty and compared fibrinogen levels between patients with and without persistent infections. The diagnostic accuracy of fibrinogen in this study was similar to the one found in our study (AUC: 0.773 vs. 0.769). Moreover, Xu et al. also found that fibrinogen’s diagnostic accuracy was higher than that of D-dimer (AUC: 0.565), which is also in line with our findings (AUC: 0.628). Last, Pannu et al. assessed the diagnostic accuracy of D-dimer for persistent joint infection prior to reimplantation in 53 patients [22]. The authors reported that the diagnostic accuracy of D-dimer was low (AUC = 0.620), similar to the AUC value of D-dimer in our study (AUC = 0.628). Overall, the results of these studies indicate that fibrinogen has a higher accuracy for detecting persistent infections among other coagulation-related biomarkers, probably due to its role as an acute phase protein.

The evidence regarding the diagnostic role of viscoelastic studies in PJI is scarce. A recent study by our group evaluated the diagnostic accuracy of ROTEM analysis for PJI in 65 patients who underwent revision total hip and total knee arthroplasty due to PJI or aseptic loosening [25,26]. The findings of this study indicated that development of PJI was associated with higher EXTEM MCF, and that the combined diagnostic accuracy of elevated EXTEM MCF and CRP is improved compared to that of CRP alone. In another study, Yuan et al. conducted a retrospective study comparing thromboelastography (TEG) parameters between 39 patients with aseptic loosening and 23 patients with PJI. The authors of this study found that a TEG parameter called maximum amplitude (MA) had the highest accuracy among all TEG parameters for PJI [27]. Interestingly, the TEG parameter MA is similar to the ROTEM parameter MCF, which was found to have the highest diagnostic accuracy for persistent joint infections in our study.

There are certain limitations in this study. First, the population of this study is relatively small. However, this is the first study that, to the best of our knowledge, assessed the diagnostic accuracy of ROTEM parameters for persistent infection prior to reimplantation in two-stage exchange arthroplasties. Second, the comparison between the accuracy of ROTEM parameters and that of inflammatory markers such as ESR and CRP for persistent infection might be biased, since one of the prerequisites for reimplantation is a downward trend in ESR and CRP levels. Therefore, the results of this comparison should be interpreted with caution. Third, other inflammatory markers such as procalcitonin were not measured; therefore, the diagnostic accuracy of these markers was not compared to that of ROTEM parameters.

## 5. Conclusions

The success of two-stage exchange arthroplasty for the treatment of PJI relies on complete infection eradication prior to reimplantation. Unfortunately, this is not always ensured as there are not any reliable biomarkers for detection of persistent infection prior to reimplantation. In search of new diagnostic modalities for PJI, the bi-directional cross talk between infection and coagulation can be proved to be a valuable diagnostic strategy. In line with this, our findings indicate that ROTEM parameters have the potential to identify persistent infections prior to reimplantation in two-stage exchange arthroplasties for PJI, since a higher FIBTEM MCF value was associated with a higher risk for persistent joint infection, with a high detection accuracy. Development of such coagulation biomarkers would be of great value as they could provide guidance to surgeons, along with other clinical and laboratory findings, regarding the optimal timing for reimplantation, decreasing the risk of subsequent revision surgeries due to an insufficiently treated PJI. However, this is a pilot study with a small population, while further studies with larger populations are needed to validate the diagnostic accuracy of ROTEM analysis for persistent periprosthetic joint infections.

## Figures and Tables

**Table 1:** Demographics and clinical characteristics of the study population (*n* = 70).

	With Persistent Infection (*n* = 6)	Without Persistent Infection (*n* = 64)	*p*-Value
Age (years)	66.6 ± 6.6; 64.5 (62–68)	68.5 ± 6.2; 70 (66.5–72)	0.14
Gender (males, %)	3 (50.0)	27 (42.1)	0.51
BMI (kg/m^2^)	22.8 ± 2.0; 22.5 (21–25)	24.4 ± 3.6; 24 (22–26)	0.28
CCI	4.3 ± 1.5; 4 (3–5)	4.0 ± 1.4; 4 (3–5)	0.72
Smoking (%)	2 (33.3)	4 (6.2)	0.07
Operated joint (THA, %)	4 (66.6)	39 (60.9)	0.57
Anticoagulants (%)	2 (33.3)	16 (25.0)	0.48
Antiplatelets (%)	1 (16.6)	9 (14.0)	
Vitamin K antagonists (%)	0 (0.0)	1 (1.5)	
NOACs (%)	1 (16.6)	6 (9.3)	

Data are presented as mean ± SD, median and interquartile range (IQR), or as absolute frequency (percentage) when appropriate. The non-parametric Wilcoxon rank-sum test and the chi-squared test were used for comparison between groups. Abbreviations: BMI, Body Mass Index; CCI, Charlson Comorbidity Index; THA, Total Hip Arthroplasty; NOAC, Novel Oral Anticoagulant.

**Table 2:** Conventional laboratory assays of the study population (*n* = 70).

Variables	With Persistent Infection (*n* = 6)	Without Persistent Infection (*n* = 64)	*p*-Value
PLTs (×10^3^/mL)	261.8 ± 58.1; 264.0 (216.0–295.0)	270.1 ± 43.4; 271.0 (238.0–298.0)	0.67
aPTT (s)	33.1 ± 3.8; 32.5 (30.0–34.0)	32.3 ± 4.9; 32.0 (30.0–34.2)	0.58
PT (s)	13.1 ± 2.0; 13.5 (12.0–14.0)	12.5 ± 2.1; 12.3 (11.7–14.0)	0.46
D-dimers (mg/L)	0.6 ± 0.3; 0.8 (0.5–0.9)	0.6 ± 0.7; 0.4 (0.1–0.8)	0.29
Fibrinogen (mg/dL)	415.0 ± 73.7; 413.0 (348.0–476.0)	334.0 ± 79.2; 330.0 (293.5–380.0)	**0.029**
CRP (mg/L)	5.5 ± 0.6; 5.4 (5.2–6.2)	12.1 ± 15.9; 3.9 (3.3–17.0)	0.31
ESR (mm/h)	12.6 ± 6.9; 15.5 (4.0–17.0)	12.2 ± 12.5; 12.0 (2.30–18.0)	0.38

Data are presented as mean ± SD or median and interquartile range (IQR). The nonparametric Wilcoxon rank-sum test was used for comparison between groups. Abbreviations: PLTs, platelets; aPTT, activated partial thromboplastin time; PT, prothrombin time; CRP, C-reactive protein; ESR, erythrocyte sedimentation rate. Bold indicates statistical significance at *p* value < 0.05.

**Table 3:** ROTEM parameters (prior to reimplantation) in patients with or without persistent periprosthetic joint infection.

Variables	With Persistent Infection (*n* = 6)	Without Persistent Infection (*n* = 64)	*p*-Value
EXTEM CT (s)	72.3 ± 1.9; 72.0 (71.0–74.0)	74.5 ± 33.1; 70.0 (64.0–75.0)	0.38
EXTEM CFT (s)	85.8 ± 10.3; 83.5 (78.0–88.0)	81.1 ± 33.1; 85.0 (60.5–91.0)	0.52
EXTEM A10 (mm)	61.0 ± 4.1; 61.0 (57.0–64.0)	51.7 ± 12.1; 52.0 (45.0–59.5)	**0.030**
EXTEM MCF (mm)	72.3 ± 6.1; 73.0 (68.0–74.0)	60.8 ± 11.9; 62.0 (54.0–69.5)	**0.010**
EXTEM Alpha angle	72.6 ± 8.5; 73.5 (64.0–81.0)	70.9 ± 5.7; 72.0 (68.0–75.0)	0.65
EXTEM LI60 (%)	92.6 ± 1.3; 92.5 (92.0–93.0)	93.1 ± 5.1; 93.0 (90.0–97.5)	0.65
INTEM CT (s)	182.8 ± 5.4; 185.0 (180.0–187.0)	186.6 ± 37.5; 183.5 (166.0–196.5)	0.96
INTEM CFT (s)	70.3 ± 6.9; 69.5 (65.0–75.0)	75.1 ± 52.1; 69.0 (50.5–79.5)	0.75
INTEM A10 (mm)	64.0 ± 4.3; 63.0 (62.0–68.0)	52.3 ± 10.5; 53.0 (47.0–59.0)	**0.004**
INTEM MCF (mm)	70.3 ± 4.3; 70.5 (68.0–74.0)	60.2 ± 9.5; 60.5 (54.5–67.5)	**0.006**
INTEM Alpha angle	77.1 ± 12.2; 75.0 (70.0–80.0)	75.1 ± 5.7; 76.5 (70.0–79.5)	0.98
INTEM LI60 (%)	91.8 ± 4.7; 92.0 (90.0–95.0)	90.5 ± 4.7; 92.0 (88.0–94.0)	0.59
FIBTEM CT (s)	49.1 ± 3.3; 48.5 (48.0–50.0)	50.0 ± 5.1; 50.0 (47.0–52.0)	0.48
FIBTEM A10 (mm)	17.3 ± 5.2; 17.5 (12.0–19.0)	11.6 ± 5.2; 11.0 (8.5–15.0)	**0.023**
FIBTEM MCF (mm)	24.5 ± 7.4; 22.5 (18.0–32.0)	13.4 ± 5.6; 13.5 (9.0–17.0)	**0.001**

Data are presented as mean ± SD or median and interquartile range (IQR). The non-parametric Wilcoxon rank-sum test was used for comparison between groups. Abbreviations: CT, clotting time; CFT, clot formation time; A10, clot amplitude at 10 min; MCF, maximum clot firmness; LI60, lysis index at 60 min. Bold indicates statistical significance at *p* value < 0.05.

**Table 4:** Results of multivariable logistic regression analyses for persistent periprosthetic joint infection (dependent variable) with ROTEM parameters, age, sex, BMI, Charlson comorbidity index, smoking status, and chronic anticoagulants included in the models, as independent variables.

Variables	Persistent Infection		
	OR	95% CI	*p* Value
EXTEM A10	1.11	0.98–1.25	0.09
EXTEM MCF	1.21	1.02–1.43	**0.026**
INTEM A10	1.22	1.03–1.46	**0.030**
INTEM MCF	1.27	1.02–1.57	**0.026**
FIBTEM A10	1.29	1.004–1.66	**0.044**
FIBTEM MCF	1.30	1.04–1.63	**0.020**
Fibrinogen	1.01	1.006–1.03	**0.041**
D-dimer	0.95	0.27–3.35	0.94

Abbreviations: OR, odds ratio; CI, confidence interval; A10, clot amplitude at 10 min; MCF, maximum clot firmness. Bold indicates statistical significance at *p* value < 0.05.

**Table 5:** Accuracy of ROTEM parameters and conventional studies for persistent infection.

Parameter	Persistent Infection			
	AUC (95% CI)	Optimal Cut-Off	Sensitivity (%)	Specificity (%)
EXTEM A10	0.768 (0.648–0.881)	≥57	100.0	60.9
EXTEM MCF	0.816 (0.667–0.965)	≥65	100.0	54.6
INTEM A10	0.856 (0.745–0.967)	≥63	83.3	81.2
INTEM MCF	0.837 (0.701–0.973)	≥98	100.0	60.9
FIBTEM A10	0.830 (0.666–0.994)	≥17	66.6	85.9
FIBTEM MCF	0.907 (0.812–1.000)	≥18	100.0	73.4
Fibrinogen	0.769 (0.587–0.952)	≥340	100.0	43.7
D-dimer	0.628 (0.426–0.831)	≥0.6	83.3	54.6

Abbreviations: AUC, area under curve; CI, confidence interval; A10, clot amplitude at 10 min; MCF, maximum clot firmness.

## Data Availability

The raw data supporting the conclusions of this article will be made available by the authors on request.
